# Nobiletin Alleviates Ferroptosis-Associated Renal Injury, Inflammation, and Fibrosis in a Unilateral Ureteral Obstruction Mouse Model

**DOI:** 10.3390/biomedicines10030595

**Published:** 2022-03-03

**Authors:** Yi-Hsin Lo, Shun-Fa Yang, Ching-Chang Cheng, Kuo-Chiang Hsu, Yu-Syuan Chen, Yu-Ya Chen, Chun-Wei Wang, Siao-Syun Guan, Cheng-Tien Wu

**Affiliations:** 1 Department of Nutrition, China Medical University, Taichung 406040, Taiwan; u108076007@cmu.edu.tw (Y.-H.L.); kchsu@mail.cmu.edu.tw (K.-C.H.); u108008441@cmu.edu.tw (Y.-S.C.); luf870707@gmail.com (Y.-Y.C.); u109076010@cmu.edu.tw (C.-W.W.); 2 Institute of Medicine, Chung Shan Medical University, Taichung 40201, Taiwan; ysf@csmu.edu.tw; 3 Department of Medical Research, Chung Shan Medical University Hospital, Taichung 40201, Taiwan; 4 Laboratory Animal Service Center, Office of Research and Development, China Medical University, Taichung 40402, Taiwan; chengjzvm@mail.cmu.edu.tw; 5 Master Program of Food and Drug Safety, China Medical University, Taichung 406040, Taiwan; 6 Institute of Nuclear Energy Research, Atomic Energy Council, Taoyuan 32546, Taiwan; ssguan@iner.gov.tw

**Keywords:** chronic kidney disease, ferroptosis, fibrosis inflammation, nobiletin

## Abstract

Nobiletin (Nob), a critical active flavonoid of citrus fruits, has received attention for its superior physical functions, which have shown to improve the progression of diseases. Chronic kidney disease (CKD) is recognized as a global health problem, and its mortality and morbidity rates are worsened with an increased risk of accompanying disorders. In this study, we aimed to elucidate whether Nob treatment ameliorates kidney fibrosis and also to identify the potential signaling networks in a unilateral ureteral obstructive (UUO) mouse model, which was used to mimic the progression of CKD. Six-week-old C57BL/6J mice were orally treated with 50 mg/kg of Nob for 14 constitutive days after UUO surgery. We found that the administration of Nob diminished kidney fibrosis and the expression of EMT markers, ameliorated oxidative stress and ferroptosis-associated injury, and mitigated the inflammatory response in the kidneys of UUO mice. Our results suggested that Nob treatment has antiferroptosis, anti-inflammatory, and antifibrotic effects, improving the progression of CKD in UUO mice. Nob may serve as a potential therapeutic candidate for the improvement of progressive CKD in further studies.

## 1. Introduction

Chronic kidney disease (CKD) is a global health problem and had morbidity and mortality rates of around 40% between 1990 and 2017 [1]. In 2017, according to global statistical reports, the prevalence of CKD was 9.1%, representing approximately 700 million cases of all-stage CKD and 1.2 million deaths because of irreversible end-stage renal disease (ESRD) or the combined effects of other diseases, such as hypertension and cardiovascular complications [1,2]. This evidence suggests an area of unmet need regarding the improvement of the progression of CKD and the importance of the discovery of novel targeted treatments. Renal fibrotic development is a critical feature, involving biological and pathological changes of the kidney, inflammatory complications, and renal function loss in CKD lesions. It is considered a final common pathway to ESRD [3]. During this process, several active factors, such as transforming growth factor-β (TGF-β), α-smooth muscle actin (α-SMA), nuclear factor-κB (NF-κB), and NADPH Oxidase 4 (NOX4), contribute to a chronic inflammatory response, oxidative stress injury, extracellular matrix (ECM) accumulation, and renal fibrosis [4,5,6]. Active NF-κB could directly or indirectly trigger a proinflammatory response, attract leucocyte infiltration [7], and accelerate the expression of TGF-β in kidney tubular epithelial cells [8]. Induced TGF-β could also promote the expression of α-SMA, which is a renal fibrotic marker, and also causes the accumulation of ECM and the deposition of collagen [9]. This signaling network in the progression of CKD is complicated, and effective therapeutic strategies to restore or protect against ESRD in clinical patients remain unavailable.

Ferroptosis, a kind of programmed necrotic cell death, is now regarded as a key target to participate in kidney diseases [10,11]. Borawski et al indicated that the maintained iron homeostasis effectively prevents acute kidney injury (AKI) and may provide a new therapeutic intervention for AKI [12]. Indeed, a recent pilot study also showed the improvement of AKI by the inhibited the ferroptosis with quercetin treatment [13]. Furthermore, accumulation studies indicated the involvement of ferroptosis stress and signaling in the regulation of inflammatory response, cell death, and fibrosis through AKI to CKD transition [14]. As exemplified by these recent evidence suggested that ferroptosis may play a role in the progression of CKD.

Nob, a unique dietary class of flavonoids in the peel of citrus fruits [15], displays low-toxicity and multiple pharmacological properties, including anti-inflammatory, inhibition of oxidative stress, immunomodulatory, anti-cancer, neuroprotective, antiatherosclerosis, and antidiabetic effects [16,17]. More recently, Liu et al. demonstrated the antiapoptotic effects of Nob treatment in myocardial infarction and fibrosis in a rat model via JNK regulation [18]. Li et al. demonstrated that Nob treatment mitigates inflammation and apoptotic death in a murine model [19]. Xu et al. reported the protective effects of Nob administration on diabetic nephropathy via the inhibition of NF-κB. This evidence suggests the potential therapeutic benefits of Nob treatment for the improvement of progressive diseases. However, whether Nob treatment ameliorates the progression of CKD remains to be clarified.

In this study, therefore, we explored the potential therapeutic efficacy of Nob treatment and molecular signaling with a unilateral ureteral obstructive (UUO) mouse model. The UUO model is a stable and well-established model for determining expression changes of apoptotic cell death, inflammation, oxidative stress injury, and fibrosis symptoms in abnormal kidneys [20].

## 2. Materials and Methods

### 2.1. Animal Care, Nob Treatment, and UUO Surgical Administration

Six-week-old male C57BL/6J mice were purchased from the Laboratory Animal Center (NLAC), NARLabs, Taiwan, and treated under the guidance of the Laboratory Experimental Animal Center of China Medical University (CMU). Animal care and welfare, UUO administration, and the treatment protocol (plan ID: CMUIACUC-2020-263-2) were approved by the Animal Research Committee of CMU. The mice were humanely housed with food and water ad libitum in a Specific Pathogen-Free (SPF) room. The temperature was maintained at 22 ± 2 °C with a 12 h light and dark cycle. After a week of acclimation, the mice were randomly divided into sham, UUO, and Nob treatment groups (*n* = 8); subjected to a surgical UUO procedure to cause chronic kidney injury; and then orally treated with 50 mg/kg Nob (AdooQ BioScience, Irvine, CA, USA) for 14 days. Candesartan (5 mg/kg, Sigma-Aldrich, St. Louis, MO, USA), a RAS inhibitor, has been demonstrated as therapeutically effective for CKD and was used as the positive control [21,22]. UUO administration was performed as in our previous study [21,23]. After Nob treatment for adaptive time points, the mice were anesthetized with isoflurane and necropsied, and UUO and contralateral kidneys were isolated.

### 2.2. Histopathological Detection

The isolated kidneys of the UUO mice were fixed in 4% formaldehyde saline buffer for one week. After paraffin embedding, 4-micrometer-thick kidney sections were used to detect histopathological changes by Hematoxylin and Eosin (H&E) staining (Sigma-Aldrich, St. Louis, MO, USA). The score for renal injury levels was described in our previous study [24]. Briefly, renal sections were deparaffinized and dehydrated by immersion in 70%, 85%, and 95% ethanol saline buffer for 5 min, and then we stained the nucleus and cytoplasm of the renal sections with hematoxylin and eosin buffer (Sigma-Aldrich, St. Louis, MO, USA). Renal injury scoring was assessed based on the pathological changes of the cortex areas, including tubular dilation, necrotic tubular cells, glomerular tuff, sclerosis, and inflammatory cell infiltrations from 15 random fields. Abnormal areas were evaluated using a blind test with scores of 0 = normal; 1 = mild (<25%, abnormal pathology injury); 2 = moderate (25–50%); 3 = severe (50–75%); and 4 = large area injury (>75%). For Mason’s trichrome staining, 4-micrometer-thick rehydrated renal sections were stained with Bouin’s fluid, Weigert’s iron hematoxylin working solution, and aniline blue solution, as in the manufactory protocol (Sigma-Aldrich, St. Louis, MO, USA). Collagen deposition areas showed blue staining, and they were quantified by Fovea Pro 4.0 imaging software (Reindeer Graphics, Asheville, NC, USA).

### 2.3. Western Blotting Analysis

Kidney samples from the sham control, UUO, and treatment groups were homogenized, and then we quantified the total amount of protein using a bicinchoninic acid (BCA) assay (Thermo Fisher Scientific Inc., Bremen, Germany). The expression of protein levels was performed by Western blotting, as in our previous study [25]. Briefly, a total of 20 μg proteins were identified and loaded into the sodium dodecyl sulfate (SDS) polyacrylamide gel (8–15%). Subsequently, SDS polyacrylamide gel transferred all proteins to the PVDF membrane (Millipore Technology, Billerica, MA, USA), which were then blocked with 5% serum albumin/Tris-buffered saline (TBST) solution and incubated with primary antibodies overnight at 4 °C, including fibronectin (1:500 dilution; BD Biosciences, San Jose, CA, USA); α-SMA (1:1000 dilution; Sigma−Aldrich, St. Louis, MO, USA); collagen I, E−cadherin, TGF−beta, TrxR1, NOX4, TFR1, GPx4, SLC7A11/xCT, Bax, Bcl−2, Pro−caspase 3, and phosphorylated NFκB−p65 (p−p65) (1:1000 dilution; Cell Signal Technology, Danvers, MA, USA); and a total form of NFκB−p65, SOD−2, catalase Pro-caspase 3, cyclooxygenase−2 (COX−2), and beta-actin (1:1000 dilution; Santa Cruz Biotechnology, Santa Cruz, CA, USA). After transferring to the secondary antibody (1:5000 dilutions; Santa Cruz Biotechnology, Santa Cruz, CA, USA), the changes in the protein samples were detected by an enhanced chemiluminescence kit (Millipore Technology, Billerica, MA, USA) in a digital photo-image system (Azure Biosystem, Inc., Dublin, CA, USA).

### 2.4. Immunohistological Staining

The 4-micrometer-thick kidney sections were used to assess inflammatory cell infiltration, as described previously [23]. Briefly, sections were deparaffinized at 65 °C, rehydrated with 90%, 75%, and 50% ethanol/saline buffer, and then boiled for antigen retrieval. After blocking in 5% fetal bovine albumin (FBS; Thermo Fisher Scientific Inc., Bremen, Germany), sections were incubated in a 3% hydrogen peroxide/methanol solution to deplete peroxidase, and then Ly6g (1:200 dilution; eBioscience, San Diego, CA, USA) and F4/80 (1:200 dilution; Cell Signal Technology, Danvers, MA, USA) primary antibodies were used to detect the expression areas of neutrophils and macrophages, respectively. Finally, sections were color-performed with the Polymer-HRP linker and a 3,3′-diaminobenzidine tetrahydrochloride detection kit (BioGenex, Fremont, CA, USA), and positive staining areas were quantified with Fovea Pro imaging software (version 4.0, Adobe system Inc., San Jose, CA, USA).

### 2.5. Fluorescent Terminal Deoxynucleotidyl Transferase dUTP Nick End Labeling (TUNEL) Staining

Renal apoptotic cells of UUO mice were detected by fluorescent TUNEL staining, as Liu et al. described [25]. Briefly, renal sections were deparaffinized and rehydrated in 50–90% ethanol/saline buffer, incubated in protease K, and then the apoptotic cells were stained with a fluorescent TUNEL assay kit (Promega Inc., Madison, WI, USA). Counterstaining was performed using Hoechst 33258 (Sigma-Aldrich, St. Louis, MO, USA). After mounting all sections, TUNEL-positive staining cells were detected and calculated from 15 randomly selected fields using fluorescence microscopy under 400× magnification.

### 2.6. Real-Time Quantitative Polymerase Chain Reaction

Kidney samples from UUO mice were isolated, sliced, and homogenized in TRIzol reagent (Sigma-Aldrich, St. Louis, MO, USA). The total amount of 15 μ RNA was reverse transcribed to the reaction volume of 50 μL with a mix reverse transcriptase reagent (Promega Inc., Madison, WI, USA). The RT product was quantified, and 10 ng RT primers were added, including GAPDH (forward, TGGCACAGTCAAGGCTGAGA; reverse, CTTCTGAGTGGCAGTGATGG); 18S (forward, AGTCCCTGCCCTTTGTACACA; reverse, CGATCCGAGGGCCTCACTA); TNF-alpha (forward CTCAGCCTCTTCTCATTCCTG; reverse GTTTGCTACGACGTGGGCTAC); IL-6 (forward, TACAGGCTCCGAGATGAACAAC; reverse, TGCCGTCTTTCATTACACAGGA); and SYBR Green PCR amplification reagent. The gene expression of cDNA was normalized by GAPDH and 18s in the iQ5 system (Bio-rad, Hercules, CA, USA).

### 2.7. Statistical Analysis

The results are presented as mean ± S.D. A *p*-value of <0.05 was considered to indicate a significant difference between the sham control and treatment groups. Data were analyzed by one-way analysis of variance (ANOVA) with the post hoc Tukey HSD. SigmaPlot (version 12.0, Systat Software, Inc., San Jose, CA, USA) software was used in this study.

## 3. Results

### 3.1. Nob Treatment Ameliorates Pathological Changes and Renal Fibrosis in the Kidneys of UUO Mice

**Figure 1:**
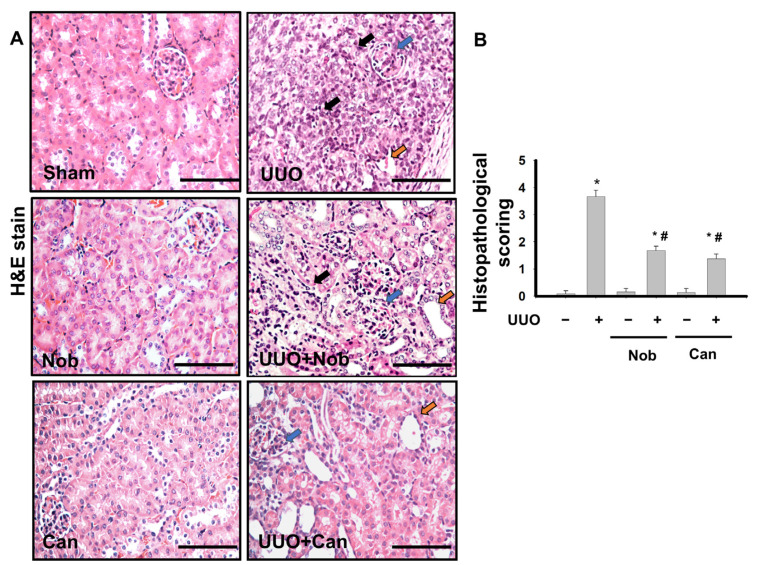
Nob treatment ameliorates renal pathological changes in a UUO mouse model. C57BL/6J mice were orally administrated a vehicle, Nobeltin (Nob), or Candesartan (Can) for 14 consecutive days after UUO surgery. The pathological changes were observed by H&E staining. The features of kidney injury, including glomerular Tuff (blue arrow), tubular dilation (yellow arrow), and inflammatory cells infiltration (black arrow), are shown. Magnification: 400×; Scale bar: 50 μm (**A**). Renal injury scoring is shown in (**B**). Data are presented as mean ± SD (*n* = 6). * *p* < 0.05 vs. the sham control group. # *p* < 0.05 vs. the UUO group.

**Figure 2:**
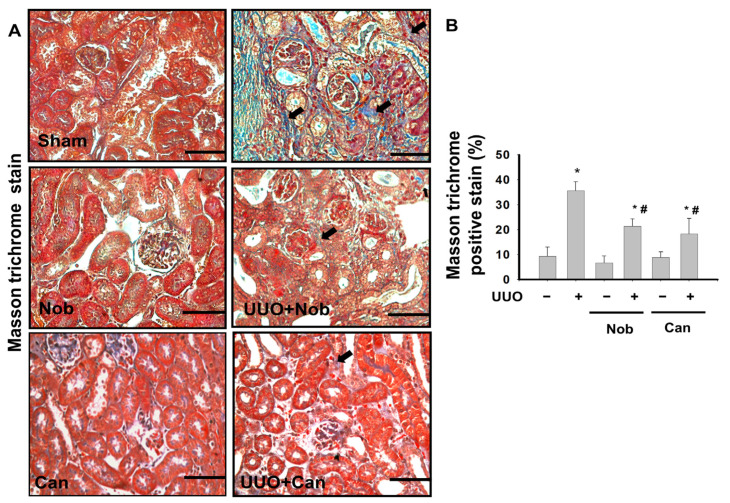
Administration of Nob improves collagen deposition in the UUO kidney. Vehicle, Nob, or Candesartan were orally administered for 14 days to UUO mice. Collagen deposition (black arrow) was determined by Masson’s Trichrome staining. Magnification: 400×; Scale bar: 50 μm; a. Control; b. Nob; c. Can; d. UUO; e. UUO+Nob; f. UUO+Can. (**A**). Quantification of collagen deposition is shown in (**B**). Data are presented as mean ± SD (*n* = 6). * *p* < 0.05 vs. the sham control group. # *p* < 0.05 vs. the UUO group.

Renal fibrosis is the most important hallmark of pathological changes in the progression of CKD [26]. To test whether Nob treatment reduced renal pathological changes, H&E staining was performed after UUO administration on Day 14. As shown in Figure 1, control, Nob, and Can (positive control) kidney sections were not significantly different. However, some pathological injuries, including leukocyte infiltration (black arrow), tubular dilation (yellow arrow), and glomerular Tuff (blue arrow), were revealed in UUO kidneys. On the contrary, mild complications were observed in the UUO+Nob and positive-treatment groups. Similarly, we also found severe collagen deposition (black arrow, blue staining) in the kidney of UUO mice, which was significantly attenuated by Nob or Can administration (Figure 2).

**Figure 3:**
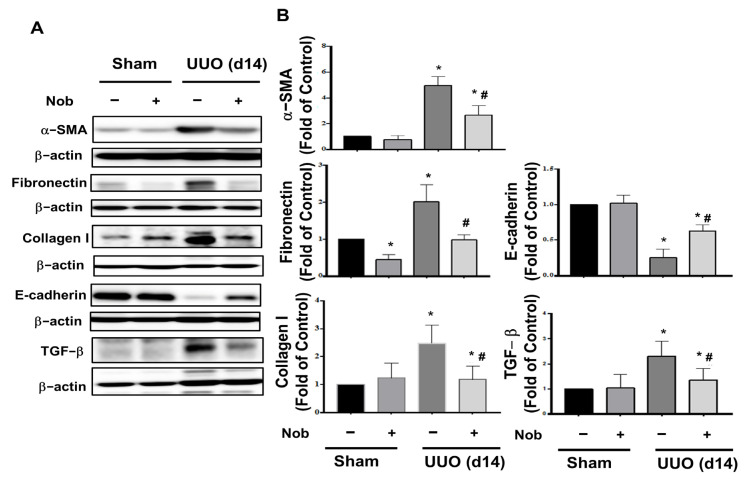
Nob treatment attenuates protein expression of renal fibrosis and EMT markers in the kidneys of UUO mice. C57BL/6J mice were orally administrated vehicle or Nob for 14 consecutive days after the UUO model process. Protein expression of fibrosis and EMT markers, including α-SMA, Fibronectin, Collagen I, E-cadherin, and TGF-beta, were analyzed by Western blotting (**A**). Quantification of protein levels is shown in (**B**). Data are presented as mean ± SD (*n* = 6). * *p* < 0.05 vs. the sham control group. # *p* < 0.05 vs. the UUO group.

Epithelial-to-mesenchymal transition (EMT) of tubular epithelial cells is one of the most important characteristics of progressive fibrosis [27]. Therefore, we assessed the protein expression levels of fibrosis markers, including α-SMA, collagen, and fibronectin [28], as well as EMT markers, including TGF-beta and E-cadherin [29,30], using Western blotting analysis. As shown in Figure 3, the kidney of the surgical UUO groups showed significantly elevated protein expressions of α-SMA, collagen, fibronectin, and TGF-beta, while E-cadherin was diminished. After Nob treatment, these fibrotic and EMT markers were conspicuously reversed. These results suggested that oral treatment with Nob prominently improves renal pathological changes and the progression of renal fibrosis in UUO mice.

### 3.2. Administration of Nob Attenuates Oxidative Stress Injury, Ferroptosis, and Apoptotic Cell Death in the UUO Kidney

Oxidative stress induction played an important role in the apoptotic cell death and progression of renal fibrosis of UUO mice [6,31]. Interestingly, some recent evidence has indicated that the dysregulation of oxidative stress could also contribute to ferroptosis-induced cell death in disease conditions, including kidney injury [32,33]. Therefore, we then interpreted the protective effects of Nob treatment on oxidative stress and ferroptosis in the UUO kidney of mice.

**Figure 4:**
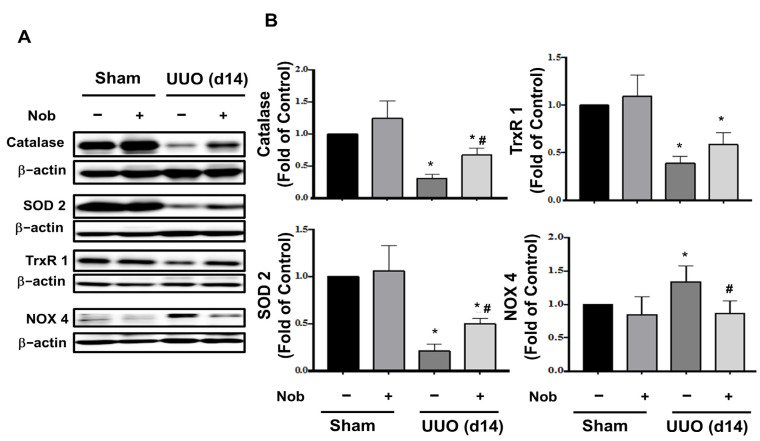
Nob treatment mitigates oxidative stress injury in the UUO kidney. C57BL/6J mice were orally treated with vehicle or Nob and the protein expression levels of oxidative stress-associated antioxidant enzymes, including catalase, SOD-2, TrxR1, and Nox4, were analyzed using Western blotting analysis (**A**). The quantification is shown in (**B**). Data are presented as mean ± SD (*n* = 6). * *p* < 0.05 vs. the sham control group. # *p* < 0.05 vs. the UUO group.

As shown in Figure 4, protein expression levels of the antioxidant enzymes, including catalase, superoxide dismutase 2 (SOD 2), and thioredoxin reductase 1 (TrxR1), a regulator of Nrf2 [34], were significantly suppressed, while Nox4, a member of the NADPH oxidase family, which is involved in the development of renal fibrosis [25,35], was significantly augmented in UUO kidneys. After oral treatment with Nob, these disturbed protein expressions were reversed in UUO kidneys.

**Figure 5:**
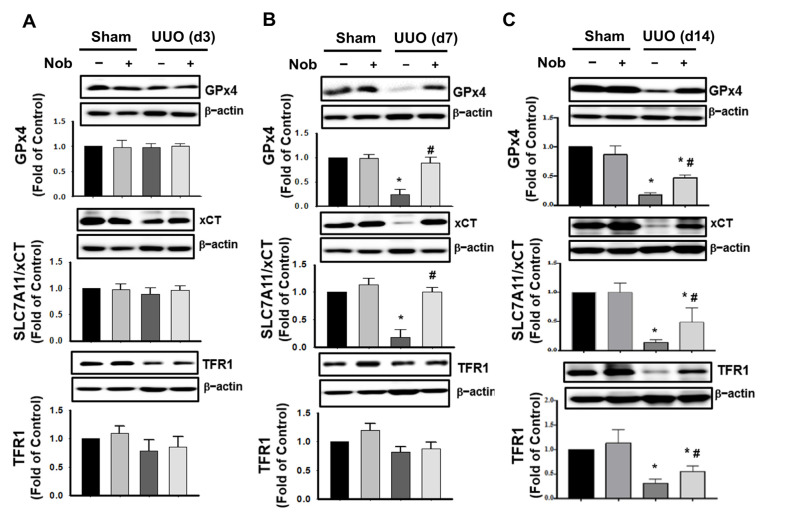
Nob treatment restores protein depletion during the progression of ferroptosis. C57BL/6J mice were orally treated with vehicle or Nob and the protein expression levels of ferroptosis markers, including GPx4, SLC7A11/xCT, and TFR1, were assessed at Day 3 (**A**); Day 7 (**B**); and Day 14 (**C**) by Western blotting analysis. Data are presented as mean ± SD (*n* = 6). * *p* < 0.05 vs. the sham control group. # *p* < 0.05 vs. the UUO group.

Some empirical data have indicated that glutathione peroxidase 4 (GPx4), Solute carrier family 7 member 11 (SLC7A11/xCT) [36,37], and transferrin receptor (TFR1) are the markers of ferroptosis [38]. Next, we observed the changes of ferroptosis protein expression levels during the progress of CKD mice. After treatment with Nob for 3 days, the protein expression of GPx4, SLC7A11/xCT, and TFR1 were not significantly changed in the kidney of UUO mice (Figure 5A). However, GPx4 and SLC7A11/xCT were dramatically downregulated at Day 7, and all protein markers could be protected against obstruction-induced depletion by Nob treatment by Day 14 (Figure 5B,C). These results suggested that oral Nob treatment attenuated oxidative-stress induced injury and also mitigated the induction of ferroptosis in the kidneys of UUO mice.

### 3.3. Administration of Nob Prevents Renal Apoptotic Cell Death in UUO Mice

**Figure 6:**
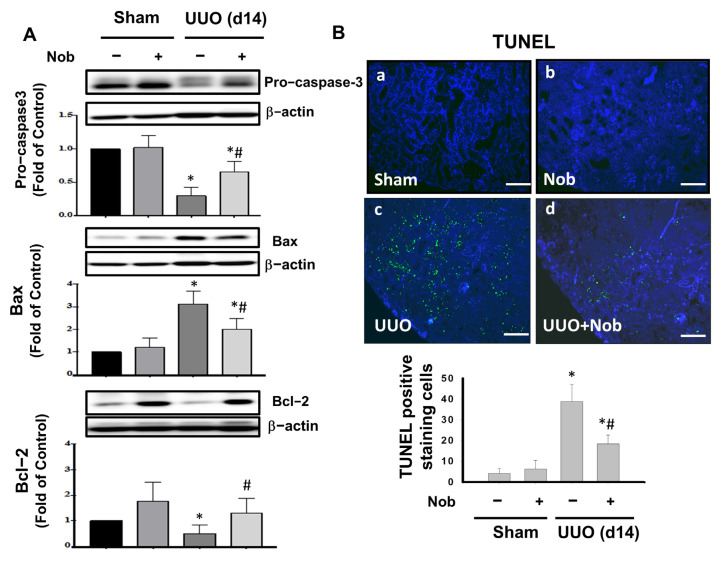
Administration of Nob reduces apoptotic renal cell death in UUO mice. C57BL/6J mice were orally treated with vehicle or Nob for 14 days after UUO surgery. Protein expression of apoptotic death markers, including pro-caspase 3, Bax, and Bcl-2, is shown in (**A**). Fluorescent TUNEL staining (**B**). Magnification: 200×; Scale bar: 100 μm; a. Control; b. Nob; c. UUO; d. UUO+Nob. Quantification of TUNEL positive cells is shown in (**B**). Data are presented as mean ± SD (*n* = 6). * *p* < 0.05 vs. the sham control group. # *p* < 0.05 vs. the UUO group.

Apoptosis is an important pivotal event for the onset of progressive renal fibrosis [39]. Moreover, Sulistiyowati et al. indicated that apoptosis and Bcl-2-associated X protein (Bax) and B-cell lymphoma-2 (Bcl-2) signaling are implicated in the development of fibrosis in UUO mice. We then determined whether Nob treatment prevents apoptotic cell death in the kidneys of UUO mice. As shown in Figure 6A, the protein expression of pro-caspase 3 and Bcl-2 were conspicuously reduced, while Bax was significantly elevated, in the kidney of UUO mice at Day 14. After orally administrating Nob for 14 consecutive days, these apoptosis markers were prominently reversed in the kidneys of the UUO+Nob group. Similarly, Nob treatment also reduced renal cell apoptotic death, as shown by fluorescent TUNEL staining. These results suggested that Nob treatment could prevent renal cell apoptotic death via the regulation of Bax, Bcl-2, and caspase 3 signaling.

### 3.4. Nob Treatment Mitigates Inflammatory Cells Infiltration in the Kidneys of UUO Mice

**Figure 7:**
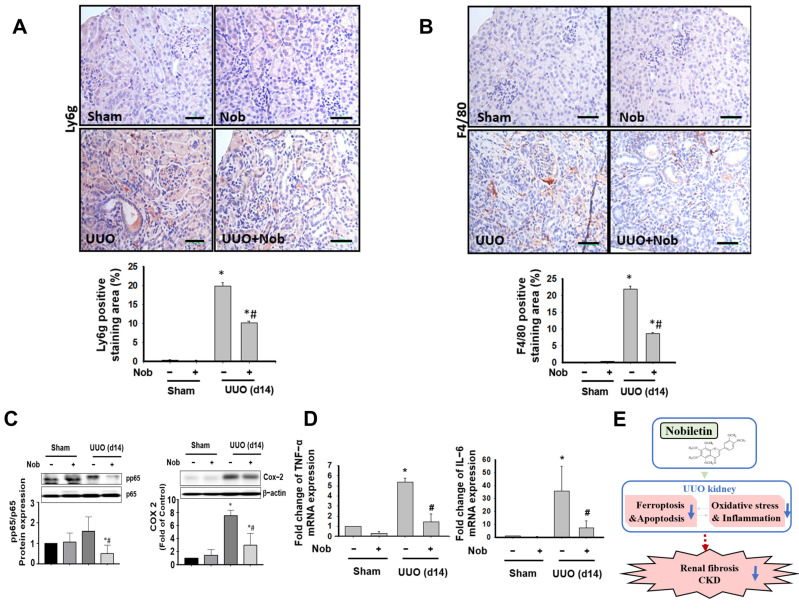
Nob treatment alleviates leucocyte cell infiltration in the kidneys of UUO mice. C57BL/6J mice were orally treated with vehicle or Nob for 14 days after UUO surgery. Neutrophil infiltration markers were detected in kidney samples by IHC staining with Ly6g (**A**) or macrophage markers by F4/80 (**B**). Magnification: 400×; Scale bar: 50 μm. Protein expression of inflammation markers, including nuclear factor-kappa b (NF-κb) and cyclooxygenase-2 (COX-2), is shown in (**C**). mRNA expression of inflammatory markers including TNF-α and IL-6 is shown in (**D**). A schematic representation of the proposed signaling networks affected by Nob treatment in CKD progression in a UUO mouse model. Blue arrow: the decreased response. Red dotted arrow: the potential process of CKD (**E**). Data are presented as mean ± SD (*n* = 6). * *p* < 0.05 vs. the sham control group. # *p* < 0.05 vs. the UUO group.

Persistent inflammation has been demonstrated to cause renal fibrosis, EMT, and progressive chronic kidney disease [40]. We investigated the effects of Nob treatment on leukocyte infiltration and the inflammatory response in the kidneys of UUO mice. As shown in Figure 7A,B, Ly6g, a neutrophil marker [41], and F4/80, a macrophage marker [42], were significantly elevated in the kidneys of UUO mice, which could be reduced by an oral administration of Nob for 14 consecutive days. Furthermore, the inflammatory signal molecules, including phosphorylated NF-κB-p65 and COX-2 protein expression, were prominently elevated following surgical UUO administration in mice, which could be alleviated after oral Nob (Figure 7C). Similarly, the induced TNF-α and IL-6 mRNA expressions in UUO kidneys were also ameliorated by the administration of Nob. These results suggested that the protective effects of Nob treatment decreased neutrophil and macrophage infiltration and inhibited the inflammation response in a UUO mouse model.

## 4. Discussion

The UUO mice model has been suggested as a stable and well-established model for active compound or drug selection. It displays the progression of obstructive nephropathy to final renal fibrosis. Several pathological features, including acute infiltration of leukocytes and the inflammatory response, glomerulosclerosis, brush border loss, tubular dilation, cell death of proximal tubular and glomerulus, and interstitial fibrosis, were observed [43]. (2) However, this model also presented certain limitations and cannot fit all features of progressive CKDs. It does not influence the blood pressure of the model animals, there is no clear proteinuria, and it is not easy to detect changes of the renal impairment parameters from serum biochemistry detection, such as BUN and creatinine, in the model due to compensation by the contralateral normal kidney [44]. On the other hand, we also found that another study has indicated that some potential urine protein markers may still be sensitive to the renal pathological changes, despite the fewer alternations of BUN, serum creatinine, and protein urea in UUO mice [45]. We will also want to detect and build higher sensitive markers or systems such as cystatin C to dissect renal pathological changes and assess the efficacy of the pharmaceutical treatment in further studies. So far, the UUO model is still regarded as high-throughput and easily operated CKD in a mouse model that can be induced with a relatively short procedure and which serves as a common model for analyzing treatments for renal fibrosis [20].

Nob, an active flavonoid of citrus fruits, has been demonstrated to aid in the improvement of LPS-induced acute lung injury by the suppression of the inflammation response in a rat model [46], inhibition of hepatic oxidative stress and fibrosis in an obese mouse model [19], protection against cardiac dysfunction in diabetic mice [47], and alleviation of acute ischemia/reperfusion in the kidney of mice [48] or following streptozotocin (STZ)-induced diabetic nephropathy in rats [49]. These results suggest potential therapeutic benefits of Nob treatment for the prevention of the progression of CKD and renal fibrosis. In this study, therefore, 50 mg/kg b. w. of Nob was selected to orally treat a mouse UUO model for 14 consecutive days. Li et al. provided Nob (50 mg/kg) or vehicle by daily intraperitoneal injection for 4 weeks to obese mice. They found protective effects against hepatocyte apoptotic death, inflammation, and fibrosis but not for the induction of an adverse response [19]. Similarly, Wang et al. also demonstrated that oral 50 mg/kg Nob for 5 weeks significantly alleviated the chronic hepatic inflammatory response and subsequent liver fibrosis in mice [50]. Another study demonstrated that the intraperitoneal use of Nob at up to 100 mg/kg for 7 consecutive days prevented ischemia/reperfusion (I/R) injury by inhibiting reactive oxygen species production and apoptosis in mice [48]. Another report indicated that oral pre-treatment with 5–20 mg/kg Nob improved LPS-induced acute lung injury and the inflammatory response by inhibition of the NF-κB signaling pathway. In this study, we orally administrated 50 mg/kg for 14 consecutive days, which effectively prevented oxidative stress induction (Figure 4) and apoptotic cell death (Figure 6), inhibited leukocyte infiltration and inflammation, (Figure 7), and reduced subsequent renal fibrosis (Figure 2 and Figure 3). Our current results and other reports may provide some empirical data supporting the possible protective effects against the progression of CKD of the oral treatment of 20 to 50 mg/kg of Nob in a UUO mouse model.

Nob treatment has been shown to have antioxidant, anti-inflammatory, anti-fibrosis, and anti-apoptotic properties, and to protect against injury in several disease models, including renal ischemia reperfusion insult [48], acetaminophen-induced hepatorenal toxicity [51], and diabetic cardiomyopathy [47]. On the other hand, the renal injury process of UUO mice has been summarized as initially involving tubular damage (oxidative stress, apoptosis), then a prolonged acute and chronic inflammatory response, followed by causing final interstitial fibrosis and glomerulosclerosis [52]. In this study, indeed, we cannot accurately point to which Nob treatment was first effective, and this can be considered as another limitation. Interestingly, however, we noted that Nob treatment, which possessed superior antioxidant action, may also protect against oxidative stress-induced injury, including programmed apoptotic death and ferroptosis during the progress of CKD.

Ferroptosis is a kind of programmed necrotic cell death, and it is now regarded to participate in kidney diseases such as sepsis, diabetic kidney disease (DKD), crystal-induced kidney disease, and allograft injury [11,53]. Recently, although more researchers have focused on ferroptosis and pathophysiological changes and the process of AKI, studies on ferroptosis and CKD remain limited [10,33]. Zhang et al. recently found an improvement of collagen deposition, cell death, and lipid peroxidation using a ferroptosis inhibitor, liproxstatin-1 (Lip-1), in a UUO mouse model, which suggests the potential involvement of ferroptosis in the progression of CKD [54]. In addition, the overload of oxidative stress is known as a key initial regulator in the progression of ferroptosis. The depleted or dysregulated expression of antioxidant enzymes such as Gpx4 and TrxR1 and ferroptosis-associated cascade proteins, such as Xct and TFR-1, could cause functional loss or impairment of organs or cells in the progression of diseases [55]. Interestingly, oxidative stress dysregulation is also observed in the UUO of CKD mice. In this study, we found that the antioxidant enzyme (Gpx4) and the interacting proteins of ferroptosis (Xct and TFR1) were significantly reduced after oral treatment with Nob on Day 7 and Day 14 (Figure 5). Furthermore, we also found that the oral administration of Nob prevented programmed apoptotic cell death (Figure 6). Our current results and other empirical evidence suggested that the administration of Nob, an active antioxidant flavonoid, may prevent programmed necrotic death (ferroptosis) and programmed apoptotic death during the progression of CKD in mice.

Renal fibrosis is an important feature in the progression of CKD. A previous pilot study considered the impact of total flavonoids of Lichi Semen on CCl4-induced liver fibrosis in rats. In that study, Nob was regarded as one of the potential targets to prevent liver injury and liver fibrosis [56]. Li et al. further demonstrated that the antioxidant, anti-inflammatory, and antifibrotic properties of Nob treatment prevented high-fat diet-induced liver injury in a rat model [19]. Another study also demonstrated that Nob treatment alleviated renal fibrosis and dysfunction, which could suppress the TGF-β1 expression of the kidneys [57]. Interestingly, Nob has been found to directly inhibit TGF-β1 signaling networks in human non-small cell lung cancer cells [58]. In this study, we found that the consecutive treatment of Nob not only suppressed the protein expression of ECM, including collagen I or fibronectin, but also, more importantly, diminished the TGF-β expression in the UUO kidney. Together with our results, these reports suggest Nob may possess antifibrotic therapeutic benefits through the inhibition of TGF-β in disease models, including the progression of CKD.

## 5. Conclusions

In conclusion, our results provide a novel perspective and key insight into the improvement of progressive CKD via Nob treatment in a UUO mouse model. Administration of Nob not only ameliorates oxidative stress injury, which is potentially associated with ferroptosis signaling and renal cell apoptotic death, but also attenuates leukocyte infiltration and EMT networks to final fibrosis, as the schematic representation of the proposed signaling shows in Figure 7D. Taken together, these results suggest that orally administrated Nob, at least in part, decreased oxidative stress and its associated ferroptosis or apoptosis, suppressed the inflammation response, and ameliorated renal fibrosis. Nob may serve as a potential therapeutic candidate for the improvement of progressive CKD in further studies.

## Data Availability

The data presented in this study are available from the corresponding author upon reasonable request.

## References

[1] GBD Chronic Kidney Disease Collaboration (2020). Global, regional, and national burden of chronic kidney disease, 1990–2017: A systematic analysis for the Global Burden of Disease Study 2017. Lancet.

[2] Carney E.F. (2020). The impact of chronic kidney disease on global health. Nat. Rev. Nephrol..

[3] Zeisberg M., Neilson E.G. (2010). Mechanisms of tubulointerstitial fibrosis. J. Am. Soc. Nephrol..

[4] Andrade-Oliveira V., Foresto-Neto O., Watanabe I.K.M., Zatz R., Camara N.O.S. (2019). Inflammation in Renal Diseases: New and Old Players. Front. Pharmacol..

[5] Branton M.H., Kopp J.B. (1999). TGF-beta and fibrosis. Microbes Infect..

[6] Dendooven A., Ishola D.A., Nguyen T.Q., Van der Giezen D.M., Kok R.J., Goldschmeding R., Joles J.A. (2011). Oxidative stress in obstructive nephropathy. Int. J. Exp. Pathol..

[7] Mukhin N.A., Kozlovskaia L.V., Bobkova I.N., Rameev V.V., Chebotareva N.B., Plieva O.K., Shcherbak A.V., Varshavskii V.A., Golitsina E.P. (2004). The key role of tubulointerstitium remodeling in progression of chronic renal diseases. Arch. Pathol..

[8] Nogueira A., Pires M.J., Oliveira P.A. (2017). Pathophysiological Mechanisms of Renal Fibrosis: A Review of Animal Models and Therapeutic Strategies. In Vivo.

[9] Meng X.M., Nikolic-Paterson D.J., Lan H.Y. (2016). TGF-beta: The master regulator of fibrosis. Nat. Rev. Nephrol..

[10] Wang J., Liu Y., Wang Y., Sun L. (2021). The Cross-Link between Ferroptosis and Kidney Diseases. Oxid. Med. Cell Longev..

[11] Belavgeni A., Meyer C., Stumpf J., Hugo C., Linkermann A. (2020). Ferroptosis and Necroptosis in the Kidney. Cell Chem. Biol..

[12] Borawski B., Malyszko J. (2020). Iron, ferroptosis, and new insights for prevention in acute kidney injury. Adv. Med. Sci..

[13] Wang Y., Quan F., Cao Q., Lin Y., Yue C., Bi R., Cui X., Yang H., Yang Y., Birnbaumer L. (2021). Quercetin alleviates acute kidney injury by inhibiting ferroptosis. J. Adv. Res..

[14] Carney E.F. (2021). Ferroptotic stress promotes the AKI to CKD transition. Nat. Rev. Nephrol..

[15] Huang H., Li L., Shi W., Liu H., Yang J., Yuan X., Wu L. (2016). The Multifunctional Effects of Nobiletin and Its Metabolites In Vivo and In Vitro. Evid. Based Complement Alternat. Med..

[16] Wu Y., Cheng C.S., Li Q., Chen J.X., Lv L.L., Xu J.Y., Zhang K.Y., Zheng L. (2021). The Application of Citrus folium in Breast Cancer and the Mechanism of Its Main Component Nobiletin: A Systematic Review. Evid. Based Complement Alternat. Med..

[17] Braidy N., Behzad S., Habtemariam S., Ahmed T., Daglia M., Nabavi S.M., Sobarzo-Sanchez E., Nabavi S.F. (2017). Neuroprotective Effects of Citrus Fruit-Derived Flavonoids, Nobiletin and Tangeretin in Alzheimer’s and Parkinson’s Disease. CNS Neurol. Disord. Drug Targets.

[18] Liu Z., Gao Z., Zeng L., Liang Z., Zheng D., Wu X. (2021). Nobiletin ameliorates cardiac impairment and alleviates cardiac remodeling after acute myocardial infarction in rats via JNK regulation. Pharmacol. Res. Perspect..

[19] Li S., Li X., Chen F., Liu M., Ning L., Yan Y., Shang Z., Huang S., Tu C. (2021). Nobiletin mitigates hepatocytes death, liver inflammation, and fibrosis in a murine model of NASH through modulating hepatic oxidative stress and mitochondrial dysfunction. J. Nutr. Biochem..

[20] Martinez-Klimova E., Aparicio-Trejo O.E., Tapia E., Pedraza-Chaverri J. (2019). Unilateral Ureteral Obstruction as a Model to Investigate Fibrosis-Attenuating Treatments. Biomolecules.

[21] Chiang C.K., Hsu S.P., Wu C.T., Huang J.W., Cheng H.T., Chang Y.W., Hung K.Y., Wu K.D., Liu S.H. (2011). Endoplasmic reticulum stress implicated in the development of renal fibrosis. Mol. Med..

[22] Higashi K., Oda T., Kushiyama T., Hyodo T., Yamada M., Suzuki S., Sakurai Y., Miura S., Kumagai H. (2010). Additive antifibrotic effects of pioglitazone and candesartan on experimental renal fibrosis in mice. Nephrology.

[23] Chen H.A., Chen C.M., Guan S.S., Chiang C.K., Wu C.T., Liu S.H. (2019). The antifibrotic and anti-inflammatory effects of icariin on the kidney in a unilateral ureteral obstruction mouse model. Phytomedicine.

[24] Wu C.T., Sheu M.L., Tsai K.S., Chiang C.K., Liu S.H. (2011). Salubrinal, an eIF2alpha dephosphorylation inhibitor, enhances cisplatin-induced oxidative stress and nephrotoxicity in a mouse model. Free. Radic. Biol. Med..

[25] Liu S.H., Wu C.T., Huang K.H., Wang C.C., Guan S.S., Chen L.P., Chiang C.K. (2016). C/EBP homologous protein (CHOP) deficiency ameliorates renal fibrosis in unilateral ureteral obstructive kidney disease. Oncotarget.

[26] Chevalier R.L., Forbes M.S., Thornhill B.A. (2009). Ureteral obstruction as a model of renal interstitial fibrosis and obstructive nephropathy. Kidney Int..

[27] Allison S.J. (2015). Fibrosis: Targeting EMT to reverse renal fibrosis. Nat. Rev. Nephrol..

[28] Meran S., Steadman R. (2011). Fibroblasts and myofibroblasts in renal fibrosis. Int. J. Clin. Exp. Pathol..

[29] Hills C.E., Squires P.E. (2010). TGF-beta1-induced epithelial-to-mesenchymal transition and therapeutic intervention in diabetic nephropathy. Am. J. Nephrol..

[30] Docherty N.G., Calvo I.F., Quinlan M.R., Perez-Barriocanal F., McGuire B.B., Fitzpatrick J.M., Watson R.W. (2009). Increased E-cadherin expression in the ligated kidney following unilateral ureteric obstruction. Kidney Int..

[31] Kawada N., Moriyama T., Ando A., Fukunaga M., Miyata T., Kurokawa K., Imai E., Hori M. (1999). Increased oxidative stress in mouse kidneys with unilateral ureteral obstruction. Kidney Int..

[32] Yu Y., Yan Y., Niu F., Wang Y., Chen X., Su G., Liu Y., Zhao X., Qian L., Liu P. (2021). Ferroptosis: A cell death connecting oxidative stress, inflammation and cardiovascular diseases. Cell Death Discov..

[33] Hu Z., Zhang H., Yang S.K., Wu X., He D., Cao K., Zhang W. (2019). Emerging Role of Ferroptosis in Acute Kidney Injury. Oxid. Med. Cell Longev..

[34] Cebula M., Schmidt E.E., Arner E.S. (2015). TrxR1 as a potent regulator of the Nrf2-Keap1 response system. Antioxid. Redox Signal..

[35] Qiao X., Wang L., Wang Y., Su X., Qi Y., Fan Y., Peng Z. (2017). Intermedin inhibits unilateral ureteral obstruction-induced oxidative stress via NADPH oxidase Nox4 and cAMP-dependent mechanisms. Ren. Fail..

[36] Li J., Cao F., Yin H.L., Huang Z.J., Lin Z.T., Mao N., Sun B., Wang G. (2020). Ferroptosis: Past, present and future. Cell Death Dis..

[37] Lin W., Wang C., Liu G., Bi C., Wang X., Zhou Q., Jin H. (2020). SLC7A11/xCT in cancer: Biological functions and therapeutic implications. Am. J. Cancer Res..

[38] Feng H., Schorpp K., Jin J., Yozwiak C.E., Hoffstrom B.G., Decker A.M., Rajbhandari P., Stokes M.E., Bender H.G., Csuka J.M. (2020). Transferrin Receptor Is a Specific Ferroptosis Marker. Cell Rep..

[39] Yang M., Zhuang Y.Y., Wang W.W., Zhu H.P., Zhang Y.J., Zheng S.L., Yang Y.R., Chen B.C., Xia P., Zhang Y. (2018). Role of Sirolimus in renal tubular apoptosis in response to unilateral ureteral obstruction. Int. J. Med. Sci..

[40] Black L.M., Lever J.M., Agarwal A. (2019). Renal Inflammation and Fibrosis: A Double-edged Sword. J. Histochem. Cytochem..

[41] Boivin G., Faget J., Ancey P.B., Gkasti A., Mussard J., Engblom C., Pfirschke C., Contat C., Pascual J., Vazquez J. (2020). Durable and controlled depletion of neutrophils in mice. Nat. Commun..

[42] Dos Anjos Cassado A. (2017). F4/80 as a Major Macrophage Marker: The Case of the Peritoneum and Spleen. Results Probl. Cell Differ..

[43] Eddy A.A., Lopez-Guisa J.M., Okamura D.M., Yamaguchi I. (2012). Investigating mechanisms of chronic kidney disease in mouse models. Pediatr. Nephrol..

[44] Zhang Q.F. (2017). Ulinastatin inhibits renal tubular epithelial apoptosis and interstitial fibrosis in rats with unilateral ureteral obstruction. Mol. Med. Rep..

[45] Yuan Y., Zhang F., Wu J., Shao C., Gao Y. (2015). Urinary candidate biomarker discovery in a rat unilateral ureteral obstruction model. Sci. Rep..

[46] Li W., Zhao R., Wang X., Liu F., Zhao J., Yao Q., Zhi W., He Z., Niu X. (2018). Nobiletin-Ameliorated Lipopolysaccharide-Induced Inflammation in Acute Lung Injury by Suppression of NF-kappaB Pathway In Vivo and Vitro. Inflammation.

[47] Zhang N., Yang Z., Xiang S.Z., Jin Y.G., Wei W.Y., Bian Z.Y., Deng W., Tang Q.Z. (2016). Nobiletin attenuates cardiac dysfunction, oxidative stress, and inflammatory in streptozotocin: Induced diabetic cardiomyopathy. Mol. Cell. Biochem..

[48] Liu B., Deng Q., Zhang L., Zhu W. (2020). Nobiletin alleviates ischemia/reperfusion injury in the kidney by activating PI3K/AKT pathway. Mol. Med. Rep..

[49] Xu M., Wang R., Fan H., Ni Z. (2021). Nobiletin ameliorates streptozotocin-cadmium-induced diabetic nephropathy via NF-kappaB signalling pathway in rats. Arch. Physiol. Biochem..

[50] Wang S.W., Lan T., Sheng H., Zheng F., Lei M.K., Wang L.X., Chen H.F., Xu C.Y., Zhang F. (2021). Nobiletin Alleviates Non-alcoholic Steatohepatitis in MCD-Induced Mice by Regulating Macrophage Polarization. Front. Physiol..

[51] Guvenc M., Cellat M., Gokcek I., Ozkan H., Arkali G., Yakan A., Yurdagul Ozsoy S., Aksakal M. (2020). Nobiletin attenuates acetaminophen-induced hepatorenal toxicity in rats. J. Biol. Mol. Toxicol..

[52] Ucero A.C., Benito-Martin A., Izquierdo M.C., Sanchez-Nino M.D., Sanz A.B., Ramos A.M., Berzal S., Ruiz-Ortega M., Egido J., Ortiz A. (2014). Unilateral ureteral obstruction: Beyond obstruction. Int. Urol. Nephrol..

[53] Wang Y., Li Y., Xu Y. (2021). Pyroptosis in Kidney Disease. J. Mol. Biol..

[54] Zhang B., Chen X., Ru F., Gan Y., Li B., Xia W., Dai G., He Y., Chen Z. (2021). Liproxstatin-1 attenuates unilateral ureteral obstruction-induced renal fibrosis by inhibiting renal tubular epithelial cells ferroptosis. Cell Death Dis..

[55] Kajarabille N., Latunde-Dada G.O. (2019). Programmed Cell-Death by Ferroptosis: Antioxidants as Mitigators. Int. J. Mol. Sci..

[56] Feng Y.Y., Yan J.Y., Xia X., Liang J.Q., Li F., Xie T.F., Luo W.S., Feng J.F. (2020). Effect and mechanism of total flavonoids of Lichi Semen on CCl_4-induced liver fibrosis in rats, and prediction of Q-marker. Zhongguo Zhong Yao Za Zhi.

[57] Bunbupha S., Apaijit K., Maneesai P., Prasarttong P., Pakdeechote P. (2020). Nobiletin ameliorates high-fat diet-induced vascular and renal changes by reducing inflammation with modulating AdipoR1 and TGF-beta1 expression in rats. Life Sci..

[58] Da C., Liu Y., Zhan Y., Liu K., Wang R. (2016). Nobiletin inhibits epithelial-mesenchymal transition of human non-small cell lung cancer cells by antagonizing the TGF-beta1/Smad3 signaling pathway. Oncol. Rep..

